# DlgS97/SAP97, a Neuronal Isoform of Discs Large, Regulates Ethanol Tolerance

**DOI:** 10.1371/journal.pone.0048967

**Published:** 2012-11-07

**Authors:** Rajani Maiya, Seonok Lee, Karen H. Berger, Eric C. Kong, Justin B. Slawson, Leslie C. Griffith, Kogo Takamiya, Richard L. Huganir, Ben Margolis, Ulrike Heberlein

**Affiliations:** 1 Ernest Gallo Clinic and Research Center, Emeryville, California, United States of America; 2 Department of Anatomy, University of California San Francisco, San Francisco, California, United States of America; 3 Department of Biology, Brandeis University, Waltham, Massachusetts, United States of America; 4 Department of Neuroscience, Faculty of Medicine, Graduate School of Medicine, University of Miyazaki, Kiyotake, Miyazaki, Japan; 5 Department of Neuroscience and the Howard Hughes Medical Institute, Johns Hopkins University, Baltimore, Maryland, United States of America; 6 Department of Internal Medicine, University of Michigan Medical School, Ann Arbor, Michigan, United States of America; Wellcome Trust Sanger Institute, United Kingdom

## Abstract

From a genetic screen for *Drosophila melanogaster* mutants with altered ethanol tolerance, we identified *intolerant* (*intol*), a novel allele of *discs large 1 (dlg1)*. *Dlg1* encodes Discs Large 1, a MAGUK (Membrane Associated Guanylate Kinase) family member that is the highly conserved homolog of mammalian PSD-95 and SAP97. The *intol* mutation disrupted specifically the expression of DlgS97, a SAP97 homolog, and one of two major protein isoforms encoded by *dlg1* via alternative splicing. Expression of the major isoform, DlgA, a PSD-95 homolog, appeared unaffected. Ethanol tolerance in the *intol* mutant could be partially restored by transgenic expression of DlgS97, but not DlgA, in specific neurons of the fly’s brain. Based on co-immunoprecipitation, DlgS97 forms a complex with N-methyl-D-aspartate (NMDA) receptors, a known target of ethanol. Consistent with these observations, flies expressing reduced levels of the essential NMDA receptor subunit dNR1 also showed reduced ethanol tolerance, as did mutants in the gene *calcium/calmodulin-dependent protein kinase* (*caki*), encoding the fly homolog of mammalian CASK, a known binding partner of DlgS97. Lastly, mice in which SAP97, the mammalian homolog of DlgS97, was conditionally deleted in adults failed to develop rapid tolerance to ethanol’s sedative/hypnotic effects. We propose that DlgS97/SAP97 plays an important and conserved role in the development of tolerance to ethanol via NMDA receptor-mediated synaptic plasticity.

## Introduction

In humans and other organisms, repeated consumption of alcohol leads to the development of tolerance, defined as an acquired resistance to the physiological and behavioral response to a particular concentration of alcohol (reviewed in [1], [2]). The development of tolerance is a complex event associated with multiple physiological as well as functional changes. Yet, our knowledge of the mechanisms by which prolonged or repeated ethanol exposure exerts its effects in the brain to alter behavior remains limited.

The fruitfly *Drosophila melanogaster* has been developed as a useful model system to identify molecules and pathways involved in the development of ethanol tolerance [3], [4], [5], [6], [7], [8], [9], [10], [11], [12], [13], [14], [15]. We identified a mutant, which we named *intolerant* (*intol*) that exhibits greatly reduced ability to develop tolerance. The *intol* mutant was found to carry a mutation in the gene *discs large 1* (*dlg1*). The protein products of *dlg1*, Discs Large A (DlgA) and DlgS97, are the highly conserved fly homologs of the mammalian PSD-95 and SAP97 scaffolding proteins, respectively. They are involved in targeting and clustering membrane receptors, including a known ethanol target, the N-methyl-D-aspartate receptor (NMDAR), at the synapses [16].

Acute alcohol exposure antagonizes NMDAR function, while chronic alcohol consumption leads to an increase in NMDAR-mediated neurotransmission [17]. The subsequent alterations in synaptic function are hypothesized to involve changes in receptor density and phosphorylation, which are thought to impact receptor clustering and/or complex formation [18], [19], [20]. The resulting changes in glutamate receptor signaling at the synapse have been implicated in the development of ethanol dependence, tolerance, and addiction [1], [21]. Therefore, proteins that regulate these synaptic remodeling events are thought to gate the development of ethanol-induced synaptic and behavioral plasticity [1], [17].

Via alternative splicing, the *Drosophila dlg1* locus encodes two major protein products, DlgA and DlgS97, which exhibit largely similar domain structures [22], [23]. Interestingly, the mammalian isoforms of these proteins, PSD-95 and SAP97, respectively, are encoded by two separate genes [24]. Both DlgA and DlgS97 are expressed at larval neuromuscular synapses, where DlgA is important for normal development and the organization of an intricate protein network in the postsynaptic region [25]. Recently, DlgA and DlgS97 were shown to be differentially expressed during development and adulthood: only DlgA is required for adult viability, while specific loss of DlgS97 leads to perturbation of circadian activity and courtship [26]. The mammalian homolog of DlgS97, SAP97, is ubiquitously expressed in the brain and can localize to pre- and/or post-synaptic sites of excitatory or inhibitory synapses [27]. SAP97 has been shown to interact with the C-terminus of NMDA and α-amino-3-hydroxy-5-methyl-4-isoxazolepropionic acid (AMPA) receptors [28], [29], [30], [31]. Recently, SAP97 has also been implicated in trafficking of NMDARs at the cell surface by sorting them through an unconventional secretory pathway [32].

In this study, we report a novel role for DlgS97 and SAP97 in the development of tolerance to ethanol. By testing multiple independently isolated *dlg1* alleles, we determined that DlgS97 is required for the development of rapid ethanol tolerance in *Drosophila*. We further demonstrate that hypomorphic mutants of *Nmdar1*, encoding the *Drosophila* NMDA receptor 1 (dNR1) subunit, or *Camguk/Caki*, encoding the homolog of CASK that interacts with the L27 domain of DlgS97 [22], exhibit reduced ethanol tolerance. Co-immunoprecipitation of dNR1 and DlgS97 confirmed a physical interaction of DlgS97 and NMDARs. We propose that this interaction is of functional relevance to ethanol tolerance development. Lastly, we show that adult mice with reduced expression of SAP97 display deficits in the development of rapid tolerance to the sedative/hypnotic effects of ethanol, measured using regain of the loss of righting response (LORR) [33], [34], [35].

## Materials and Methods

### Ethics Statement

All animal protocols were approved by the Ernest Gallo Clinic and Research Center (EGCRC) Institutional Animal Care and Use Committee (approval number 10.01.205).

### *Drosophila* Strains and Genetic Analysis

All flies were raised and maintained on standard cornmeal molasses agar at 25°C and 70% humidity. For the behavioral screen, P-element insertion lines were generated by mobilizing the pGawB transposable element [36], [37]. The tolerance screen that led to the identification of *intolerant* (GenBank accession number: JM426603) screened a small number of strains, 42 lines, comprising a subset of this P-element insertion collection. The control line for the *intolerant* mutant was the otherwise isogenic, parental background strain, *w;iso* (*CJ1*) (2202U, [38]). For all behavioral experiments, fly lines were outcrossed for five generations to 2202U flies prior to behavioral testing. Fly strains harboring mutant alleles of *dlg1* were obtained from the following sources: *dlg1^m52^* [39]; Bloomington Stock Center); *dlg^15779^* (Bloomington Stock Center); *dlg^NP4134^, dlg^NP768^, dlg^N7P229^, and dlg^NP1102^* (GAL4 Enhancer Trap Insertion Database (GETDB)). The following *dlg1* constructs were utilized: *UAS-eGFP-dlgA* [40] and *UAS-eGFP-DlgS97* [41], which were generously provided by Dr. Uli Thomas at the Leibniz Institute for Neurobiology in Germany. The *GAL4^c747^* enhancer stock and fly stocks harboring *NMDAR1* mutant alleles, *NR1^EP331^* and *NR1^EP3511^* [42] were obtained from the Bloomington Drosophila Stock Center. The *Nmdar1* deficiency stock, *NR1^90B^.^0^* (in which the *itpR83D* IP_3_ receptor gene is also deleted) was generously provided by the laboratory of Dr. Hasan at the National Centre for Biological Sciences, India [43]. The GFP-balanced stock (90B.0/TM3-Ser-GFP) was generated by crossing homozygotic deficiency stock with GFP balancer flies (TM6b/TM3-Ser-GFP).

The P-element insertion in the *dlg1-intol* mutant was characterized using inverse PCR and DNA sequencing. Mutants of Drosophila *CASK* (*Camguk*/*Caki*) were generated by imprecise P-element excision and have been described [44].

### Ethanol Behavioral Assays

Unless otherwise stated, adult (2–4 days post-eclosion) male flies were used for all experiments. Flies were collected under CO_2_ anesthesia 2–3 days before experiments. Ethanol sensitivity and tolerance were quantified using the inebriometer, an apparatus that measures the loss of postural control with increasing time of exposure to ethanol vapor of a sample population of flies [4], [6], [45], [46]. To quantify ethanol tolerance, a sample of ∼120 flies was exposed to ethanol vapor at a relative flow rate of 60/40 ethanol vapor/humidified air for 30 min, transferred to large vials containing fly food, and allowed to recover from sedation for an additional 3.5 hr (total time of 4 hr elapsed between the first and the second exposures). In parallel, a second, control sample otherwise identically treated was exposed to humidified air without ethanol at an equivalent total flow rate and allowed to recover. Following recovery, each pair of ethanol pre-exposed and control samples were assayed using side-by-side inebriometers. Tolerance was calculated in minutes as the difference in inebriometer mean elution time (MET) between naive and ethanol-pre-exposed flies using the formula (MET_exp2_-MET_exp1_) [7].

For some experiments, where stated, sensitivity and tolerance to ethanol were measured using sedation assays, performed essentially as previously described [37]. Sample populations of ∼25–30 flies were pre-exposed to ethanol vapor or to humidified air (control) as described above, and 4 hr later were tested for loss of ability to stand upright during exposure to ethanol vapor in the Booz-O-Mat apparatus. Sedation sensitivity was quantified as the ST50, the time for 50% of the flies in a sample population of 25–30 flies to become sedated, and tolerance was calculated as the difference in ST50 between flies previously exposed to ethanol vs. control (ST50_ exp2_-ST50_ exp1_). Chronic tolerance, ethanol sedation assay and ethanol absorption and metabolism are described in supplemental methods.

### Antibodies and Immunofluorescence Microscopy

For larval NMJ staining, wandering third instar larvae were dissected in Ca^2+^-free phosphate-buffered saline (PBS), fixed in 4% paraformaldehyde in PBS for 20 min, washed several times with PBS containing 0.1% Triton X-100 (PBST), and blocked in PBST supplemented with 5% fetal bovine serum (FBS) and 0.1% bovine serum albumin (BSA, blocking buffer) for 1 hour at RT. Following blocking, larvae were incubated with primary antibodies in blocking buffer at 4°C overnight. For adult brain staining, flies were collected 1–3 days after eclosion. CNSs were dissected in PBS, fixed in 4% paraformaldehyde in PBS for 20 min at room temperature, washed three times for 10 mins in PBS containing Triton X-100 at 0.3%, blocked for 1 hour at RT in blocking buffer and incubated with primary antibodies in blocking buffer overnight at 4°C. Samples were washed three times for 10 mins in PBST at RT, and secondary antibodies were applied in blocking buffer for 2 hr at RT. After washing three times for 10 min in PBST and once in PBS, samples were mounted in Vectashield (Vector Labs, Burlingame, CA). The following primary antibodies were used: mouse anti-DLGPDZ (1∶2000, [40]) Guinea-pig anti- DlgS97N (1∶4000, the polyclonal anti- DlgS97N antibodies were generated by immunizing both rabbits and guinea-pigs with the amino-terminal 205 amino acids of dSAP97 fused to GST (Cocalico Biologicals, Inc., Reamstown, PA), rabbit anti-NR1 antibody (used at 1∶300 for the western and was generated by injection of C-terminal peptide stretch containing KTRPQQSVLPPRYSPGYTSD; ZyMed Laboratories, Inc.), mouse anti FASII antibody (1∶500, 1D4 from Developmental Studies Hybridoma Bank), mouse anti GFP antibody (1∶500, Molecular Probe) and Cy3-conjugated Goat anti-horseradish peroxidase (1∶250, Jackson ImmunoResearch). Fluorescently-labeled secondary antibodies were purchased from Molecular Probes (1∶500, Alexa-495, −568 and −650). Larval and brain images were captured using a Leica TCS SP2 confocal microscope.

### Immunoblotting and Immunoprecipitation

For protein analysis of mutant alleles, 3 larval body wall muscles or 10 adult heads per sample were homogenized in 50 µl of SDS sample buffer (InVitrogen LDS 4X buffer containing 50 mM DTT), separated by SDS-PAGE and blotted onto nitrocellulose transfer membrane. After blocking in Tris buffered saline containing 0.1% Tween (TBST) and 5% dry milk, the membrane was incubated with anti-DlgPDZ at 1∶500, anti-DlgS97N antibody at 1∶1000, or mouse anti-Tubulin antibody (Sigma, St. Louis, MO) at 1∶10,000. For immunoprecipitation of protein complexes from adult head extracts, flies were collected 2–5 days after eclosion and frozen at –80°C for at least a day. Approximately 100 µl volume of adult heads were collected via sieve and homogenized in 1 ml of RIPA buffer containing 50 mM Tris-HCl, pH7.5, 150 mM NaCl, 1 mM EDTA, 1% Nonidet P-40, 0.5% sodium deoxycholate, 0.1% SDS and protease inhibitor cocktail (purchased from Roche). Solubilization was carried out at 4°C for 60 min, followed by centrifugation at 100,000×g for 60 min at 4°C. Another round of re-solubilization was performed on the pellet after the centrifugation. The supernatant was collected, combined and reconstituted in a final volume of 4 ml of RIPA buffer with final concentration of 0.1% Triton X-100. Subsequently, this protein extract was dialyzed for 16 hr in 50 mM Tris, pH 7.5, containing 0.1% Triton X-100 at 4°C. For immunoprecipitation, 2 µl of appropriate antiserum was added to 50 µl (resuspended resin) of protein A beads (GE Healthcare) in PBT (PBS containing 0.1% Triton X-100) for at least 2 hrs at 4°C. Protein A beads were then pelleted, washed in PBT, incubated with 1 ml of the detergent-solubilized protein extract for 4 hrs at 4°C with constant rotation. The beads were then washed with 50 mM Tris, pH 7.5, containing 0.1% Triton X-100 and 150 mM NaCl. After the wash, the beads were resuspended in 4X SDS sample buffer (40 µl), heated for 15 min at 80°C, resolved by SDS-PAGE (7% gel) and transferred to a nitrocellulose membrane. Following blocking in TBST/5% dry milk, the membrane was washed in PBS and incubated in primary antibody (rabbit polyclonal anti-NR1 peptide antibody, diluted 1∶1,000) in blocking solution (TBS with 5% BSA) overnight at 4°C.

For immunoblotting mouse brain tissue, brains were isolated from mice 3–4 weeks after the final injection of Tamoxifen and homogenized in 500 µl of RIPA buffer containing protease inhibitors and incubated on ice for 1 hour. Protein estimation was performed using the BCA reagent (Pierce, Rockford, Illinois). 20–30 µg of protein was electrophoresed and transferred on to a 0.45µm PVDF membrane (Invitrogen, Carlsbad, CA). Monoclonal antibody against Dlg was from BD transduction laboratories (CA, USA). Monoclonal antibody against Actin was from Sigma (St. Louis, MO).

### Floxed *Sap97* Mice

Floxed *Sap97* mice were generated and maintained on a mixed C57B6J/129J mice in Dr. Richard Huganir’s laboratory [47] at Johns Hopkins University. For tolerance experiments, these mice were backcrossed to C57B6/J mice for 3 generations. Homozygous floxed mice were then crossed to mice carrying the ESR-Cre transgene on a C57B6J background. Heterozygous *Sap97^fl/+;cre^* mice were then crossed with *Sap97^fl/fl^* mice to generate the *Sap97^fl/fl^*, *Sap97^fl/+;cre^* and *Sap97^fl/fl;cre^* littermates used in this study. 8-week old mice were injected with 120–130 mg/kg Tamoxifen (TM, Sigma, St. Louis, MO) dissolved in 10% (v/v) ethanol solution in corn oil (Sigma, St. Louis, MO) for 4 days. Ethanol volume per injection was less than 10 µl and the last injection was given at least 3 weeks before measuring ethanol LORR.

### Rapid Ethanol Tolerance in Mice

Rapid tolerance to the sedative/hypnotic effects of ethanol was measured using LORR assay. 3–4 weeks after the last TM injection, male mice were injected with 4 g/kg ethanol (20% w/v in saline) on day 1. After ethanol injection, mice were placed on their backs. Mice were determined to have lost their righting reflex when they were unable to right themselves three times within 30 seconds. The animals were deemed to have regained their righting response when they were able to right themselves three times in 30 seconds. LORR duration was calculated as the time interval between when the righting response was lost and when it was regained. The procedure was repeated on Day 2 and duration of LORR was recorded as on Day 1. Tolerance was calculated as the difference in the duration of LORR on Day 1 and Day 2.

### Quantitative PCR

RNA was extracted from the brains of 8–12 week old male and female *Sap97^fl/fl^*, *Sap97^fl/+; cre^* ^,^ and *Sap97^fl/fl;cre^* mice 3–4 weeks after TM injection. Complementary DNA (cDNA) was generated from 1 µg of RNA using reagents from Applied Biosystems (Foster City, CA, USA). Following synthesis, cDNA was diluted 1∶10 in water. TaqMan QPCR was performed using standard thermal cycling conditions using an ABI PRISM 7900 Sequence Detection System (Applied Biosystems). Amplification reaction contained 5 µl of cDNA template, 1x PCR Master Mix, 100 nM each of forward and reverse primers and 200 nM of FAM-labeled probe in a final volume of 10 µl. Sap97 primer/probe set spanning the boundary of exon 10 and 11 of the mouse Dlg1 gene was obtained from Applied Biosystems (Assay ID: Mm01344472_m1).

### Ethanol Metabolism

One week after the LORR assay, mice were injected with 4 g/kg ethanol and tail blood samples were obtained at 30, 60, 90, 120, 180 minutes post injection. Blood samples were stored at −80°C until BEC levels were measured using an NAD-ADH enzymatic assay [48].

## Results

### Identification of *intolerant*, a Drosophila Mutant with Reduced Ethanol Tolerance

**Figure 1:**
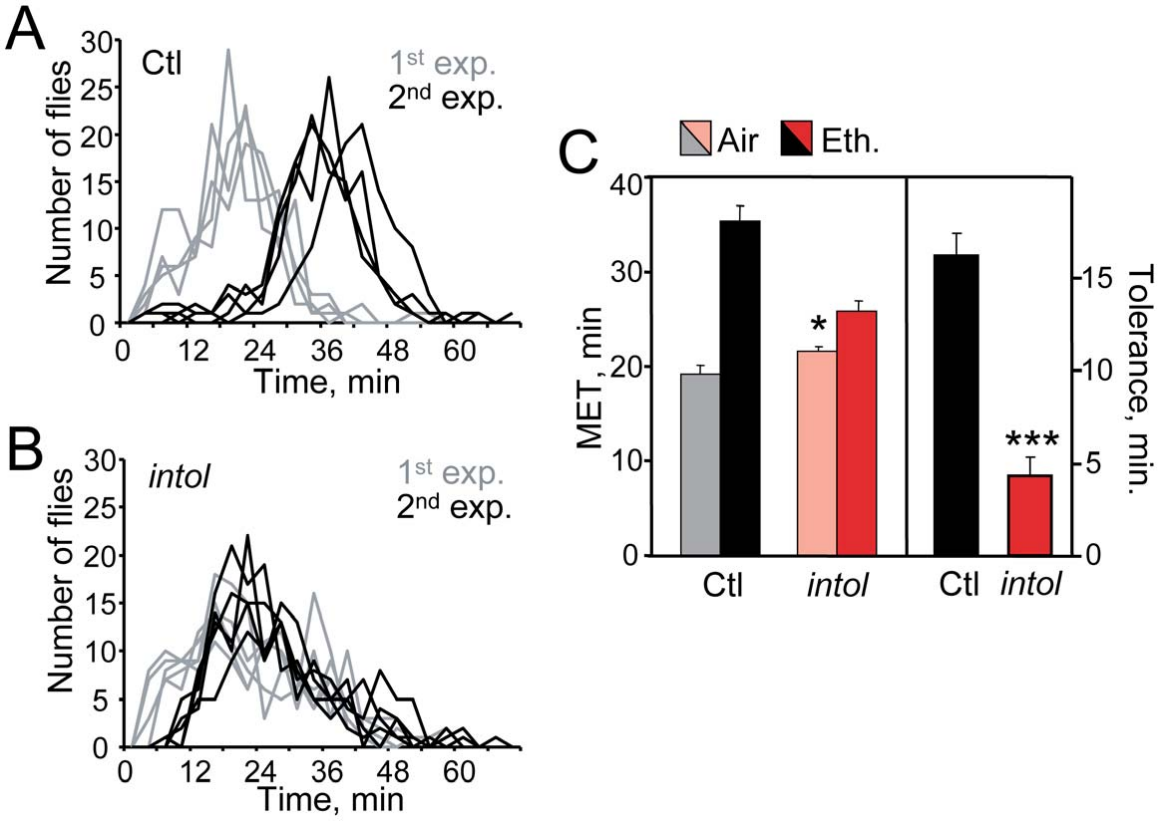
The *intol* mutant exhibits drastically reduced ethanol tolerance development. (A) Normal ethanol tolerance is displayed by control flies (parental strain, Ctl). Ethanol-naïve control flies eluted from the inebriometer with a mean elution time (MET) of 19.2±0.9 min (1^st^ exposure, gray), while the elution profiles of Ctl flies that had been exposed to ethanol 4 hr earlier, were strongly shifted to the right (2^nd^ exp., black; MET = 35.4±1.6 min). (B) Ethanol pre-exposure under otherwise identical conditions did not produce a similar increase in resistance in the *intol* mutant (1^st^ exp., gray, MET = 21.6±0.5 min; 2^nd^ exp., black, MET = 25.9±1.1 min). Inebriometer elution profiles for Ctl (A) and *intol* mutant flies (B) are shown; n = 4 (Ctl) or 5 (*intol*). Here and elsewhere, error bars represent standard error of the mean (SEM), and n refers to the number of samples, not the number of flies. (C) MET and tolerance values for Ctl and *intol* flies for inebriometer data represented in panels (A) and (B). The *intol* mutant displayed strongly reduced tolerance of 4.3±1.0 min vs. 16.2±1.2 min for Ctl flies (***, [F_(1,7)_ = 55.53, p<0.001], one-way ANOVA). Additionally, ethanol resistance in the *intol* mutant showed a modest but significant increase, with MET = 21.6±0.5 min vs. 19.2±0.9 min for Ctl flies (*, [F_(1,7)_ = 6.511, p<0.05]).

During exposure to ethanol vapor, adult flies become hyperactive, uncoordinated, and eventually sedated [49], [50]. The behavioral effects of ethanol include loss of postural control, which may readily be quantified in the inebriometer, a vertical column containing evenly spaced mesh platforms in which ∼120 flies per sample are exposed to ethanol vapor and counted as they fall from the column over time [45], [46]. Under our standard assay conditions, ethanol-naïve control flies emerged from the inebriometer with a mean elution time (MET) of ∼20 min (Fig. 1A, 1C). As previously reported, a single exposure to ethanol vapor that is sufficient to sedate flies (30 minutes at a 60/40 ratio of ethanol vapor/humidified air) leads to the development of tolerance, manifested as an increase in inebriometer MET measured 4 hours after the initial exposure, a time at which the first dose has been entirely metabolized (control flies are pre-exposed to humidified air only [4], [6]). This acquired resistance, or tolerance, correlates with an increase in the absorbed ethanol levels needed to induce loss of postural control, and has been quantified as the difference in MET between the first and second ethanol exposures [4], [7] (Fig. 1C).

To identify molecules and pathways involved in ethanol tolerance development, we carried out a small-scale genetic screen for *Drosophila* mutants exhibiting an altered response to repeated ethanol exposure. Here we report the identification of one mutant, *intolerant* (*intol*) that showed drastically reduced tolerance development compared to the parental strain (Fig. 1B, 1C). The *intol* mutant also exhibited a small but statistically significant decrease in ethanol sensitivity (Fig. 1C, left panel). Ethanol absorption and metabolism of the *intol* mutant appeared unaffected (Methods S1, Fig. S1).

Ethanol tolerance in *Drosophila* has been previously shown to be proportional to the ethanol concentration and time of pre-exposure, and requires a threshold dose [4]. We found that longer ethanol pre-exposures increased tolerance of control flies, as expected, but failed to induce tolerance in the *intol* mutant (Methods S1, Fig. S2). The *intol* tolerance defect appeared recessive, as heterozygous mutant flies developed normal tolerance (Fig. S3). The *intol* mutant was further characterized for development of chronic tolerance, a mechanistically distinct form of tolerance produced by long-term, low-level ethanol exposure [6]. In this paradigm, *intol* also exhibited reduced tolerance (Fig. S4).

### The *intol* Mutation Disrupts Expression of DlgS97, a Neuronal Isoform of Dlg1

**Figure 2:**
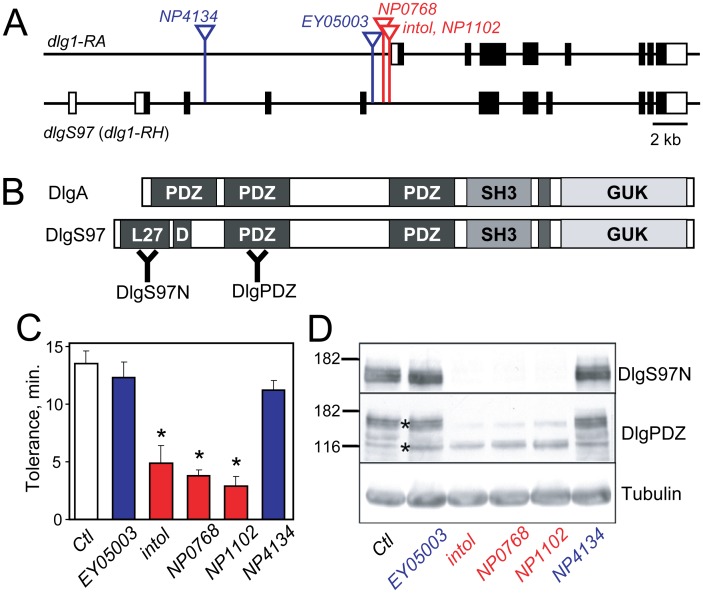
Genomic structure, additional mutants and protein products of the *dlg1* locus. (A) Schematic representation of *dlg1* genomic structure for two major characterized transcripts, *dlg1-RA* and *dlg1-RH*. Exons are represented with boxes, protein-coding sequences by filled boxes, and introns by lines. Vertical lines with inverted triangles denote independent P-element insertions in *dlg1* including *intol*. (B) Schematic depiction of DlgA (*dlg1-RA* product) and DlgS97 (*dlg1-RH* product) showing characterized protein domains. Two distinct regions used for generating antibodies are indicated as “Y” in bold. (C) Two additional transposon mutants in *dlg1*, *NP0768* and *NP1102*, whose positions are shown in red in panel (A), also showed reduced ethanol tolerance (***, [F_(5,18)_ = 19.973, p<0.001], one way ANOVA followed by Holm-Sidak test, n = 4). (D) The same subset of *dlg1* mutants showed loss of DlgS97. Immunoblot analysis was performed on protein from adult heads from control flies and the *dlg1* mutants *EY05003*, *intol*, *NP0768*, *NP1102*, and *NP4314* using pan-Dlg (“DlgPDZ”) and DlgS97-specific antibodies; tubulin was used as a loading control.

Inverse PCR and DNA sequence analysis revealed that the P element in the *intol* mutant was inserted within an intron of the gene *discs large 1* (*dlg1*) (Fig. 2A). To further investigate the involvement of *dlg1* in ethanol tolerance, we characterized the effects of additional, independently identified P element insertions in the *dlg1* locus (Fig. 2A). Ethanol tolerance was quantified for 4 additional potential *dlg1* mutants (Fig. 2C). Of these, two insertions, *NP1102* and *NP0768*, exhibited tolerance defects similar to *intol*, implying that the tolerance defects seen in these mutants is caused by the P element insertion in *dlg1*. Two other insertions, *EY05003* and *NP4134*, produced no significant decrease in tolerance compared to control flies (Fig. 2C). Similar results were obtained for these mutants in the chronic tolerance exposure paradigm (Fig. S4).

We next addressed the effect of these P element insertions on Dlg protein expression. The *dlg1* locus is complex and encodes several transcripts through use of multiple transcriptional start sites and alternative splicing (Flybase, http://flybase.org; diagrammed in simplified form in Fig. 2A and 2B). Since *dlg1* null mutations cause lethality [39], we hypothesized that the P-element mutations affecting ethanol tolerance might decrease expression of a subset of *dlg1* products. Transcripts encoded by *dlg1* are known to produce two major functional proteins, DlgA and DlgS97 (Fig. 2B), whose expression has been well characterized at the larval neuromuscular junction (NMJ) [22], [23]. DlgS97, whose protein sequence is largely shared with DlgA but contains an additional N-terminal L27 (lin-2/lin-7) heterodimerization domain [51] not present in DlgA, was shown to be the predominant form expressed in adult brain, and was implicated in circadian rhythms, phototaxis, and courtship [26]. To investigate how *dlg1* expression might relate to the ethanol tolerance defects of the mutants, protein extracts from heads of adult *dlg1* mutant and control flies were compared by western analysis using two different antibodies: one, anti-DlgPDZ, directed against a PDZ domain and thus recognizing DlgA and DlgS97 [40], and the second, anti-DlgS97N, specific for DlgS97 isoforms [41] (Fig. 2D). In protein isolated from adult fly heads, expression of DlgS97 was undetectable in the 3 mutants (*intol*, *NP0768* and *NP1102*) that displayed reduced ethanol tolerance. DlgS97 levels were unaffected in the two other mutants with normal tolerance, *EY05003* and *NP4134*. At the larval NMJ, qualitatively similar results were obtained, with DlgS97 being undetectable in the *intol*, *NP0768* and *NP1102* mutants (Fig. S5). These results show that a subset of P element insertions, including that in the *intol* mutant, disrupt expression specifically of DlgS97, and that this *dlg1-*encoded isoform is most likely required for the development of normal ethanol tolerance.

### DlgS97 Expression in the Adult Brain

**Figure 3:**
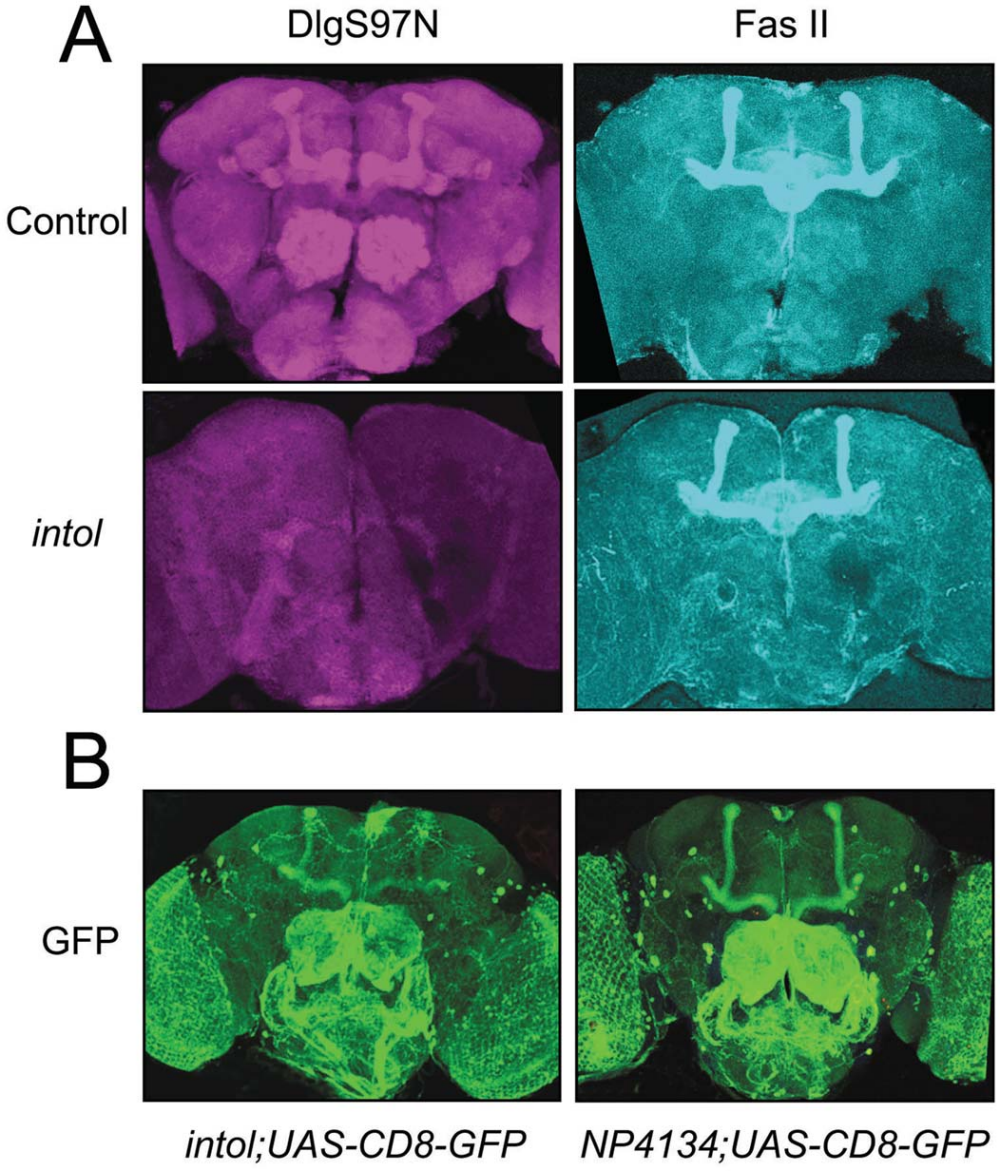
The *intol* mutant shows loss of DlgS97. (A) Whole-mount brains of Control (upper panels) and *dlg1^intol^* (lower panels) adult flies stained with anti-DlgS97 antibody (left panels, red) and anti-FASII antibody (right panels, blue). (B) Whole-mount brains of adult flies expressing UAS-CD8-GFP driven by *dlg^4134GAL4^* or *dlg^16–99GAL4^* and stained with anti-GFP antibody. Each image represents a stack of 20–25 optical sections taken at 0.5 µm steps.

Although the expression pattern and function of *dlg1* at the larval neuromuscular junction have been well elucidated, its role in the adult CNS has not been widely studied. We first determined the expression pattern of both *dlg1*-encoded isoforms, DlgA and DlgS97, in the adult brain. In wild-type adults, DlgS97 protein was prominently expressed in the mushroom body and antennal lobes (Fig. 3A), regions that are important for olfactory learning and memory [52]. This expression was abolished in *intol* mutant adult brains (Fig. 3A). In the same preparation, double-labeling with the DlgPDZ antibody (recognizing both Dlg isoforms) resulted in staining identical to that seen with the DlgS97N antibody, consistent with the finding that DlgS97 is the predominant form expressed in the adult brain [26]. Co-immunolabeling with FasII, a molecule that normally localizes to the mushroom body and central complex, revealed no gross anatomical defects in the brains of *intol* mutant flies (Fig. 3A, right panels).

The P element present in the *dlg* mutant alleles, including *intol* flies, contains the coding region for the yeast transcription factor GAL4, positioned such that GAL4 will be expressed under the control of local genomic promoters/enhancers, likely those that normally regulate Dlg expression. By crossing *intol* flies to flies carrying a *UAS-GFP* transgene, in which Green Fluorescent Protein (GFP) is expressed via GAL4-responsive *UAS* promoter elements, *dlg* enhancer-directed expression of GAL4 can be visualized. Using this method, we analyzed the potential endogenous Dlg expression pattern using two independent *dlg* alleles with different P element insertion sites. In *NP4134*, an allele that showed wild-type levels of DlgS97 protein expression and normal ethanol tolerance, GFP appeared expressed moderately in the mushroom body and strongly in the antennal lobes, subesophageal ganglion, and some additional identifiable neurons, such as the dorsal giant interneurons (DGI), neurons of the pars intercerebralis (PI) and lateral neurons (Fig. 3B, right panel). Flies in which GFP was driven by the GAL4 element of the *intol* insertion showed a GFP expression pattern similar to that seen in *NP4134;UAS-GFP* flies. However, there were some clear differences; in particular, there was a consistent reduction in GFP staining in the mushroom body, suggesting that the GAL4 element in *intol* only partially recapitulates the wild-type *dlg1* expression pattern in the adult fly brain (Fig. 3B, left panel). This has implications for experiments performed using *UAS-dlgA* and *UAS-dlgS97* transgenes to assess behavioral rescue as described below.

Both DlgA and DlgS97 are highly expressed in the post-synaptic regions of type I boutons at the NMJ ([53] and our unpublished observations). Accordingly, we examined 3^rd^ instar larval NMJs to test whether expression of DlgS97 was lost in the *dlg1* mutant larvae. In 2 different tolerance-defective mutants, *intol* and *NP1102*, DlgS97 staining appeared completely absent, whereas synaptic accumulation of DlgA persisted (Fig. S5). As a control, we examined staining in a *dlg1* null (lethal) mutant, *dlg^m52^*, at the larval NMJ and found that expression of both DlgA and DlgS97 was no longer detectable (Supplementary Fig. S5). Despite the lack of DlgS97 at the larval NMJ, electrophysiological analysis of *intol* revealed no impairment of synaptic transmission: excitatory postsynaptic potential (EPSP) amplitude, average amplitude of spontaneous miniature release events (mEPSPs), and presynaptic release, as estimated by the average EPSP/average mEPSP were normal (G. Davis, unpublished observations). These data are consistent with temporally distinct requirements for DlgA, needed for normal development, and DlgS97, needed for normal adult behavior.

### Rescue of *intol* Ethanol Tolerance Phenotype by Mushroom Body Expression of DlgS97

**Figure 4:**
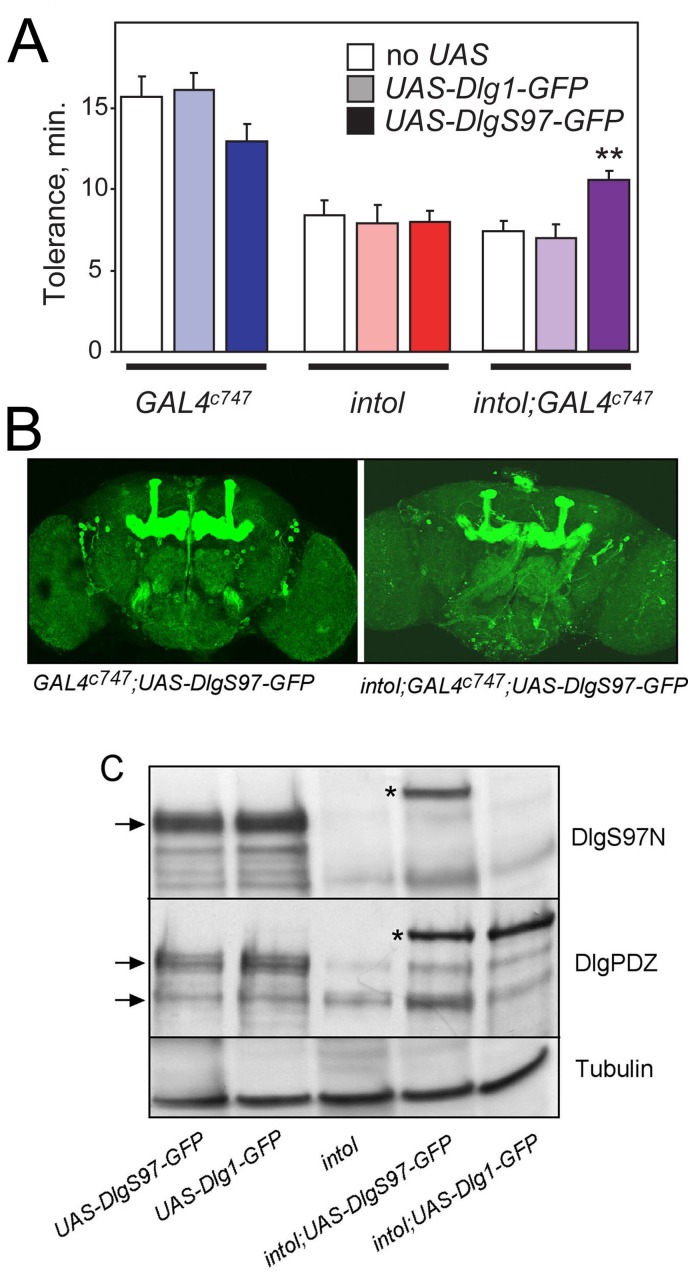
Expression of DlgS97 in a subset of neurons including in adult mushroom bodies partially rescues the tolerance deficit of the *intol* mutant. (A) Expression of DlgS97 by *GAL4^c747^* partially corrected the *intol* mutant tolerance deficit (purple bars; **, F_(2,23)_ = 7.83, P<0.01, one-way ANOVA and Holm-Sidak post hoc comparing tolerance in *intol*, *intol;UAS-Dlg1*, and *intol;UAS-DlgS97*; n = 8 or 9). Ethanol tolerance was measured using the inebriometer. A trend towards reduced tolerance was seen for (*intol^+^) GAL4^c747^* flies expressing *UAS-DlgS97*, but this difference was not significant (blue bars; p = 0.18, one-way ANOVA; n = 4–6). (B) Immunofluorescence detection of GFP in whole-mount adult brains from control and *intol* mutant flies in which expression of *UAS-eGFP-DlgS97* was driven by *GAL4^c747^*. Prominent expression is apparent in the mushroom bodies as well as elsewhere. (C) Western blot analysis detected approximately equivalent protein expression of Dlg and DlgS97 driven by *dlg^intolGAL4^*, but only UAS-DlgS97 conferred rescue of the mutant tolerance deficit.

To further investigate the involvement of DlgS97 in ethanol tolerance, we performed behavioral rescue experiments utilizing two different *dlg* transgenes. We generated wild-type and *intol* mutant flies harboring either *UAS-DlgA-GFP* or *UAS-DlgS97-GFP*. In the *intol* mutant, the transposon-encoded GAL4 appeared to direct expression of the *UAS* construct in a similar but not identical pattern to endogenous DlgS97, described above (Fig. 3). Expression of either DlgA or DlgS97 in the pattern dictated by GAL4 expression in *intol* flies did not rescue the defective ethanol tolerance of the mutant (Fig. 4A, middle bars). We verified transgene expression of DlgS97N and DlgA protein by western analysis (Fig. 4C). Both *UAS-DlgA-GFP* and *UAS-DlgS97-GFP* transgenes appeared expressed at similar levels in the *intol* background, but this level was lower than the level of endogenous expression detected in control animals (first two lanes in Fig. 4C). We next tested additional GAL4 drivers, such as *GAL4^c747^*, which is strongly expressed in the mushroom body of wild-type and *intol* flies (Fig. 4B). When *intol* mutant flies harboring *GAL4^c747^* and *UAS-DlgS97-GFP* or *UAS-DlgA-GFP* were generated and tested, we observed rescue of the mutant ethanol tolerance defect specifically with animals expressing *UAS-DlgS97-GFP*, not *UAS-DlgA-GFP* (Fig. 4A). This result further supports the hypothesis that the ethanol tolerance defect in the mutant flies is caused by loss of DlgS97 and not DlgA protein expression. The level of tolerance developed by expression of *UAS-DlgS97-GFP* in the *intol* mutant did not, however, reach that of the wild-type flies. This partial rescue might result from incomplete coverage of the relevant neurons by the *GAL4^c747^* driver. We noted, however, that *GAL4^c747^*-driven expression of *UAS-DlgS97-GFP* tended to reduce the tolerance of control flies, while expression of *UAS-Dlg1-GFP* did not (Fig. 4A), suggesting a possible dominant-negative effect caused by over-expression of DlgS97.

### NMDAR1 Hypomorphic Mutant Flies Display Reduced Ethanol Tolerance

**Figure 5:**
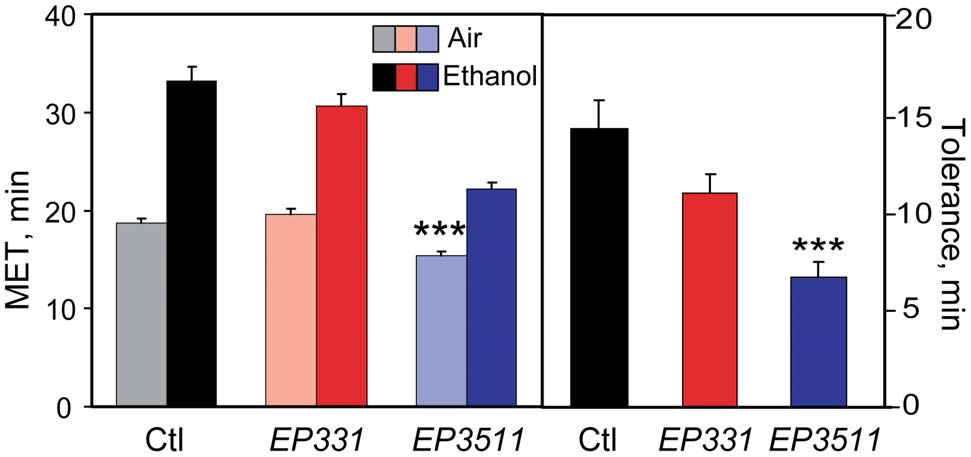
A hypomorphic mutant of *Nmdar1* exhibits reduced ethanol tolerance. Ethanol sensitivity (left panel) and rapid tolerance (right panel) were quantified using the inebriometer in 2 different homozygous insertion mutants in *Nmdar1*, *EP331* and *EP3511*, compared to control flies. The *EP3511* mutant showed a highly significant reduction in tolerance, and also exhibited increased sensitivity (***, [F_(2,20)_ = 21.338, p<0.001], one-way ANOVA with post hoc Holm-Sidak; n = 7 or 8). A second mutant in *Nmdar1*, *EP331*, showed a trend towards reduced tolerance (p = 0.057; n = 8). Dark colored bars represent inebriometer METs of flies pre-exposed to ethanol vapor, and light bars represent METs of parallel samples for each genotype pre-exposed to humidified air.

In mammals, both SAP97 and PSD95 have been shown to interact with the C-terminus of the NMDAR via their PDZ domains [28], [29]. Recent studies have shown that SAP97 plays an important role in cell surface trafficking and membrane clustering of NMDAR’s [32]. Numerous studies in rodents have demonstrated that compensatory synaptic changes occur in response to the inhibitory actions of ethanol on NMDAR complexes and that these effects may mediate synaptic plasticity involved in the development of ethanol tolerance [17]. In addition, it has been shown that NMDARs function in *Drosophila* olfactory learning and memory consolidation [42]. Hence, we hypothesized that a potential mechanism by which DlgS97 could regulate ethanol tolerance was through modulation of NMDAR function. To investigate this, we quantified ethanol tolerance in two hypomorphic mutants of *Drosophila Nmdar1*, *EP3511* and *EP331*, caused by two independent P element insertion mutations in the *Nmdar1* gene. These mutants have reduced levels of dNR1 protein, and display olfactory learning deficits [42]. Development of rapid tolerance to ethanol was significantly reduced, particularly in the *EP3511* mutant (Fig. 5). Development of chronic tolerance was likewise reduced (Fig. S6). These finding are consistent with NMDARs in flies mediating synaptic and behavioral plasticity to ethanol exposure as has been reported in mammals [17].

### Molecular Complex Formation between DlgS97 and NMDAR1

**Figure 6:**
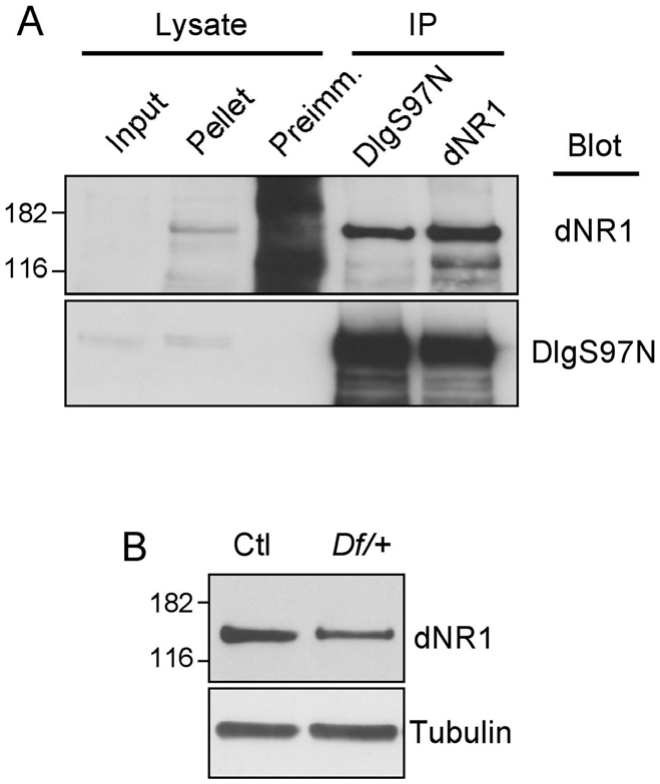
DlgS97 and NMDAR1 form a stable complex *in vivo*. (A) Immunoprecipitation of a complex containing dNR1 and DlgS97 from adult head extracts. Proteins were immunoprecipitated with pre-immune control (lane 2), anti-DlgS97 antibody (lane 3) and anti-dNR1 antibody (lane 4). Input lane represents 5% of the total lysates. (B) Western blot analysis of the anti-dNR1 antibody using control (lane 1) and dNR1 heterozygous deficiency *NR1^90B^.^0^* flies (lane 2).

**Figure 7:**
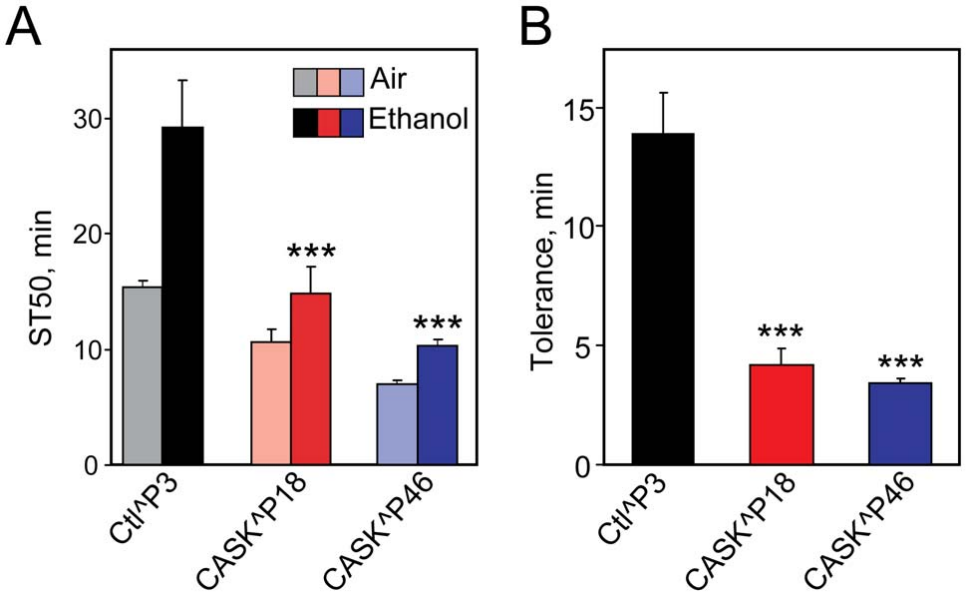
Hypomorphic mutants of Drosophila CASK display reduced ethanol tolerance. Two different hypomorphic mutants of Drosophila CASK (*CASK^P18^* and *CASK^P46^*) were tested for development of rapid tolerance, compared to an isogenic (*CASK^+^*) control. Time for 50% of each sample population of 25–30 flies to become sedated (ST50) was determined (panel A) and tolerance was quantified as the difference in ST50 between flies pre-exposed to ethanol and those pre-exposed to humidified air (panel B). There was a highly significant effect of genotype on both sensitivity and tolerance, as indicated (***, [F_(3,22)_ = 25.241, p<0.001], one-way ANOVA with post hoc Holm-Sidak; n = 6–8).

Ethanol-induced NMDAR-mediated synaptic plasticity involves synaptic remodeling that involves postsynaptic scaffolding molecules [17]. Moreover, mammalian homologs of DlgS97 interact with NMDARs and regulate subcellular targeting and synaptic plasticity mediated by NMDARs [28], [29], [54]. Therefore, we hypothesized that DlgS97 may form a complex with NMDAR1 and that its role in ethanol tolerance may be mediated through modification of NMDAR complexes in response to the acute effects of ethanol. Overall domain structures of both dNR1 and dNR2 proteins in *Drosophila* resemble those of their mammalian homologs. Sequence analysis of dNR1 revealed the presence of a type II PDZ domain binding motif, L(V)V, at the extreme C-terminus, which is absent from the dNR2 C-terminus. Hence, we focused on testing dNR1 interaction with DlgS97. We developed an anti-dNR1 antibody specific to the C-terminal domain of dNR1 (cytosolic region), that recognizes a single ∼130 kDa protein (Fig. 6B). We established the specificity of this antibody by using adult head extracts of flies heterozygous for a dNR1 deficiency, *NR1^90B^.^0^* in which the dNR1 gene is deleted [43]. The level of the putative dNR1 protein species was significantly reduced in *dNR1^90B^.^0^* heterozygotes (Fig. 6B, lane 2; homozygotes are unviable) in comparison to control flies (Fig. 6B, lane 1). We used this antibody to test whether DlgS97 formed a complex with dNR1 by immunoprecipitation of protein extracts of adult fly heads. As shown in Figure 7B, anti-dNR1 antibody, but not the cognate preimmune serum, co-precipitated DlgS97. In addition, anti-DlgS97N antibody co-precipitated dNR1, suggesting that dNR1 and DlgS97 form a complex in the adult fly brain as has been observed in the mammalian brain [31].

### Mutants in Camguk/Caki, Another Component of the Nmdar1 Scaffold, Exhibit Reduced Ethanol Tolerance

The *Drosophila* homolog of human CASK (also known as Caki or Camguk) is another L27 domain-containing molecular scaffold implicated in regulating synaptic plasticity [55]. The L27 domain of mammalian Camguk has been shown to interact with the L27 domain of SAP97 and this interaction is important for the subcellular localization of SAP97 [22]. Hence, we hypothesized that Camguk may play a role in the development of tolerance to ethanol. Mutants of *Drosophila* CASK were recently generated and characterized [44]. We tested these fly CASK hypomorphic mutants for ethanol tolerance, and found that they exhibited severely reduced tolerance, as well as increased sensitivity to ethanol sedation (Fig. 7). We suggest that the fly CASK homolog, along with DlgS97, is implicated in the adaptive molecular changes at synapses, likely involving NMDA receptor modulation, underlying the development of ethanol tolerance.

### Mice with Reduced Levels of SAP97 Display Deficits in Rapid Tolerance

**Figure 8:**
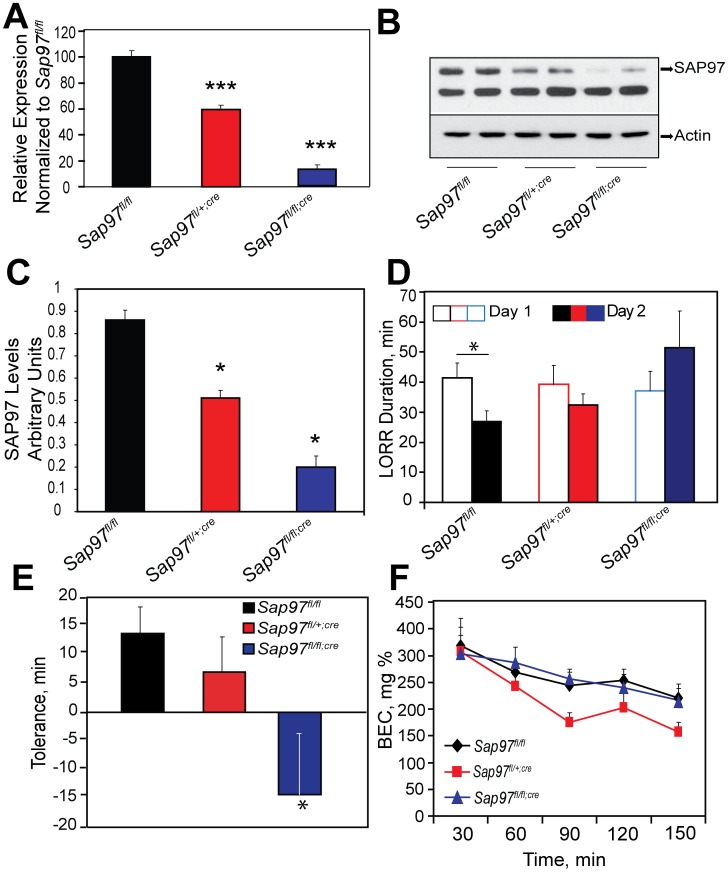
Mice with reduced SAP97 levels do not develop rapid tolerance to ethanol. (A) *Sap97* mRNA levels were compared between *Sap97^fl/fl^*, *Sap97^fl/+;cre^* and *Sap97^fl/fl;cre^* mice 3–4 weeks after TM injection. Male and female mice were used in this experiment. One Way ANOVA revealed a significant difference in *Sap97* levels between the three genotypes [F[_2_], [_15_] = 127.326, p<0.001]. Bonferroni post hoc test revealed that *Sap97* mRNA levels were significantly reduced to 60% in *Sap97^fl/+;cre^* mice (***, p<0.001) and to 10% in *Sap97^fl/fl;cre^* mice in comparison to *Sap97^fl/fl^* mice (***, p<0.001). n = 9 (*Sap97^fl/fl^*), 4 (*Sap97^fl/+;cre^*), and 5 (*Sap97^fl/fl;cre^)*. (B) Representative western blot showing reduction in SAP97 protein levels in whole brain protein extracts of *Sap97^fl/fl^*, *Sap97^fl/+;cre^* and *Sap97^fl/fl;cre^* mice 3–4 weeks after TM injection. (C) Quantification of results in B. One-Way ANOVA revealed a significant main effect of genotype [F[_2,9_] = 55.931, p<0.001]. Bonferroni posthoc test revealed that *Sap97^fl/+;cre^* and *Sap97^fl/fl;cre^* mice expressed significantly less SAP97 in comparison to *Sap97^fl/fl^* mice (***, p<0.001). n = 4/genotype. D) Ethanol-induced LORR was measured in *Sap97^fl/fl^*, *Sap97^fl/+;cre^* and *Sap97^fl/fl;cre^* mice on two consecutive days. Two-Way repeated measures (RM) ANOVA detected a significant genotype×day interaction [F[_2_], [_18_] = 4.369, p = 0.029]. Bonferroni posthoc test revealed that the duration of LORR was significantly lower on Day 2 in comparison to Day 1 in *Sap97^fl/fl^* (*, p<0.05), but not *Sap97^fl/+;cre^* or *Sap97^fl/fl;cre^* mice, n = 13 (Sap97*^fl/fl^* ), 12 (*Sap97^fl/+;cre^*), and 10 (*Sap97^fl/fl;cre^*). (E) Tolerance scores, obtained by subtracting duration of LORR on Day 1 from Day 2, are shown. One–Way ANOVA revealed a significant effect of genotype on the development of tolerance [F_(2,32)_ = 4.072, p<0.05]. Bonferroni post hoc test suggested that *Sap97^fl/fl^* mice developed significantly more tolerance to ethanol’s sedative hypnotic effects than *Sap97^fl/fl;cre^* mice, (*, p<0.05). (F) Ethanol metabolism curves are shown for the three genotypes. Two-Way RM ANOVA revealed a significant main effect of time [F_ [4,105]_ = 19.870, p<0.001] but no genotype [F_ [2,105]_ = 1.470, P>0.05] or time×genotype interaction [F_ [8,105]_ = 0.916, p>0.05] suggesting that all three genotypes metabolized ethanol at the same rate. n = 10 for (*Sap97^fl/fl^*, *Sap97^fl/fl;cre^*), n = 9 (*Sap97^fl/+;cre^*).

SAP97 is the mammalian homolog of *Drosophila* DlgS97 [24]. The loss of SAP97 function through development results in abnormalities in craniofacial development and perinatal lethality. Hence, we examined the development of rapid tolerance to ethanol in mice carrying a floxed *Sap97* allele [47]. These mice were crossed with mice carrying the estrogen receptor (ESR)-Cre transgene. In this inducible-Cre transgenic mouse line, the Cre recombinase is fused to a mutant form of the mouse ESR ligand-binding domain that does not bind endogenous ligand at physiological concentrations, but binds the synthetic ligand Tamoxifen (TM). Upon injection of TM, the Cre-ESR fusion protein translocates to the nucleus and mediates deletion of the floxed allele resulting in temporal control of gene deletion [56]. To confirm that injection of TM did indeed delete the floxed allele, we examined *Sap97* mRNA levels in the brains of *Sap97^fl/fl^*, *Sap97^fl/+;cre^* and *Sap97^fl/fl;cre^* mice 3–4 weeks after the final injection of TM. QPCR revealed that *Sap97* mRNA levels were reduced by about 50% in *Sap97^fl/+;cre^* mice and by 90% in *Sap97^fl/fl;cre^* mice (Fig. 8A) in comparison to *Sap97^fl/fl^* mice. We also examined SAP97 protein levels in whole brain protein extracts prepared from *Sap97^fl/fl^*, *Sap97^fl/+;cre^*, and *Sap97^fl/fl;cre^* mice (Fig. 8B and C). Both heterozygous and homozygous mice carrying the Cre transgene showed significantly less SAP97 expression than *Sap97^fl/fl^* mice. However, SAP97 expression was not completely eliminated even 3–4 weeks after TM injection as evidenced by the presence of a faint band corresponding to SAP97 in the *Sap97^fl/fl;cre^* mice.

We compared the development of rapid tolerance to the sedative hypnotic effects of ethanol in these mice 3–4 weeks after TM injection. Mice were injected with 4 g/kg ethanol (i.p.) and the duration of the loss of righting (LORR) was recorded on Day 1. Twenty-four hours later, these mice were again injected with the same dose of ethanol and LORR was recorded as on Day 1. Significant differences were not observed in the duration of LORR between the genotypes on Day 1, suggesting that reduced SAP97 levels did not affect sensitivity to ethanol’s sedative/hypnotic effects. The duration of LORR in *Sap97^fl/fl^* mice was 15 minutes longer on Day 2 than on Day 1 (Fig. 8D and E), suggesting the development of robust rapid tolerance to the sedative/hypnotic effects of ethanol. However, we did not observe significant differences in the duration of LORR in *Sap97^fl/+;cre^* and *Sap97^fl/fl;cre^* mice between Day 1 and Day 2, suggesting that these mice do not develop rapid tolerance to ethanol. Figure 8E shows the LORR data as a tolerance score obtained by subtracting the duration of LORR on Day 1 from Day 2. *Sap97^fl/fl;cre^* mice developed significantly more tolerance to ethanol than *Sap97^fl/fl^* mice (Fig. 8E). We also examined metabolism of 4 g/kg ethanol (Fig. 8F). Statistically significant differences were not detected in the rate of metabolism of ethanol between the genotypes, suggesting that the observed differences in rapid tolerance were not due to altered metabolism of ethanol. In summary, adult mice with reduced expression of SAP97 showed defective development of rapid tolerance to the sedating effects of ethanol, arguing for a conserved role for SAP97 in ethanol tolerance.

## Discussion

Here we describe a role for *Drosophila* DlgS97 and its murine homolog SAP97 in the development of tolerance to the sedating effects of ethanol. Our biochemical and behavioral data suggest NMDAR-mediated synaptic plasticity is a potential mechanism by which DlgS97 regulates ethanol tolerance. Furthermore, we find that mice in which the *Sap97* gene has been deleted in adults show deficits in the development of rapid tolerance to the sedative/hypnotic effects of ethanol.

### DlgS97 has a Conserved Role in Regulating Ethanol Tolerance

The Dlg-MAGUK family of scaffolding proteins, especially PSD-95 and SAP97, play critical roles in synaptic plasticity by participating in remodeling the molecular architecture at the synapse in an experience-and activity-dependent manner. As we observed, the ethanol tolerance defect of the *intol* fly mutant was only rescued upon transgenic expression of DlgS97, not DlgA, suggesting that the synaptic changes involved in developing ethanol tolerance are mediated by DlgS97.

The crucial structural difference between DlgA and DlgS97 is their N-terminus, where DlgS97 contains an L27 domain that is absent from DlgA. Biochemical and cell biological studies of the mammalian homologs of Dlg proteins, SAP97 and PSD-95, reported the importance of the L27 domain in targeting GluR1 and AMPA receptors by mediating oligomerization of SAP97 and the formation of a complex between SAP97 and PSD-95 [57], [58], [59].

The L27 domain also links SAP97 to its role in activity-dependent NMDAR and AMPAR signaling. RNAi knockdown of endogenous SAP97 reduces surface expression of both GluR1 and GluR2 and inhibits both AMPA and NMDA excitatory post synaptic currents (EPSCs) [57], demonstrating a broader role than PSD-95 in the maintenance of synaptic function. A recent study suggests that the L27 domain of SAP97 regulates synaptic plasticity by sequestering NMDARs at extrasynaptic sites [54]. The L27 domain of SAP97 is important for activity dependent regulation of AMPAR through activation of CamKII [60]. Furthermore, the L27 domain of DlgS97 interacts with Camguk and regulates CaMKII autophosphorylation, which has been shown to be a critical and evolutionarily conserved determinant of synaptic plasticity [55], [61], [62].

Consistent with the behavioral data obtained in flies, we found mice with targeted disruption of *Sap97* fail to develop tolerance to the sedative/hypnotic effects of ethanol. We chose to measure the development of tolerance to ethanol’s sedative/hypnotic effects using the LORR assay [33], [34], [35] because of the similarity of this behavioral paradigm to the one used to measure ethanol tolerance in flies. We achieved acute knockdown of SAP97 levels by crossing mice harboring the floxed *Sap97* allele with mice carrying the ESR-Cre transgene. Administering tamoxifen (TM) to these mice results in translocation of ESR-Cre into the nucleus and subsequent deletion of the floxed allele. Since TM is not administered until adulthood, we hypothesized that this approach would minimize the development of compensatory changes in the expression of other MAGUK family proteins in response to reduced SAP97 levels. Deletion of *Sap97* does not affect the duration of LORR on day 1 (ethanol-naïve mice), suggesting that SAP97 does not affect initial hypnotic sensitivity to ethanol. This finding differs from the observation that *intol* flies showed a slightly lower sensitivity to the sedating effects of ethanol (it should be noted that the *intol* mutation is not conditional; we were unable to generate an adult-specific fly *intol* mutant). *Sap97^fl/fl^* mice developed robust tolerance to the sedative/hypnotic effects of ethanol as evidenced by decreased duration of LORR on day 2 compared to day 1. In contrast, *Sap97^fl/fl^* ^;cre^ mice failed to develop rapid tolerance to ethanol’s sedative/hypnotic effects. *Sap97^fl/+;cre^* mice displayed a trend towards reduced tolerance, but these results did not reach statistical significance. In summary, these results implicate a novel and conserved role for SAP97 in mediating the development of ethanol tolerance.

### Brain Regions Implicated in Ethanol Tolerance

Previous studies showed that the ability to develop tolerance was significantly reduced in *Drosophila* mutants with structural and functional abnormalities in the central complex and mushroom body [4]. In the current study, we noted that behavioral rescue of the *intol* mutant was associated with strong expression of UAS-DlgS97 in the mushroom body. This in turn suggests that DlgS97 protein expression in the mushroom body is relevant for normal development of ethanol tolerance. Consistent with this notion, endogenous DlgS97 is highly expressed in the mushroom body. A previous study from our laboratory examined fly learning and memory “enhancer trap” mutants for their ability to develop ethanol tolerance; indeed several of the mutants with expression in the mushroom body had defective tolerance [7]. The fact that the behavioral rescue we observed for *intol; GAL4c747;UAS-DlgS97* was only partial is consistent with other brain regions also being involved, which is supported by previous studies [63].

### Molecular Interactions of DlgS97 and Ethanol Tolerance

The results of the present study indicate that in the fly brain, DlgS97 and NMDARs form a functional complex. The formation of a molecular complex between DlgS97 and NMDARs may play an important role in the clustering and subcellular targeting of NMDARs at the synapse and consequently impact synaptic plasticity and the development of tolerance to ethanol. In mammals, SAP97 is known to be involved mainly in targeting and clustering AMPARs via interaction through PDZ domains [59], [64]. However, SAP97 also binds to the NR2A and NR2B subunits of NMDA receptors [28], [29], [65]. The L27 domain of DlgS97 also interacts with Camguk, the Drosophila CASK homolog. Camguk has been shown to bind to the regulatory subunit of CaMKII in an ATP-dependent manner. Camguk modulates synaptic plasticity by regulating CaMKII autophosphorylation in an activity-dependent manner [55], [61]. The interactions of DlgS97 with Camguk and dNR1 is likely important for ethanol tolerance, given that fly Camguk and dNR1 mutants, like DlgS97 mutants, showed defective tolerance development. Our results suggest that a molecular scaffold comprised of DlgS97, Camguk, and dNR1 plays an important role in the development of tolerance to ethanol.

In summary, we have uncovered a novel role for DlgS97 in the development of tolerance to ethanol’s sedative/hypnotic effects. Strikingly, we found that mice with reduced expression of SAP97 display deficits in the development of rapid tolerance to ethanol. This further supports the notion that the molecular machinery that underlies development of tolerance to ethanol is largely conserved between flies and mammals, and identifies particular molecular players and interactions for future investigation.

## Supporting Information

Figure S1 **The** ***intolerant*** **mutant flies exhibit normal ethanol absorption and metabolism.** Control (*2202U*) and *intol* mutant flies were grown and collected as for behavioral experiments, pre-exposed to ethanol vapor or humidified air, allowed to recover and re-exposed all to ethanol vapor, and snap-frozen at time indicated by arrow in schematic. Frozen flies were processed and ethanol content quantified. No significant effect of either genotype or pre-exposure condition (air vs. ethanol vapor) was seen on ethanol content of flies, nor was there a significant interaction between genotype and pre-exposure condition (two-way ANOVA; n = 4).
(TIF)

Figure S2 **A longer ethanol pre-exposure does not correct the** ***intol*** **mutant tolerance deficit.** Parental (*2202U*) and *intol* mutant flies were pre-exposed to ethanol vapor or to humidified air (control) for 30, 40 or 50 min as indicated and allowed to recover for ∼3.5 hr. All samples were then exposed to ethanol vapor (110∶40 relative flow ethanol vapor:humidified air), and the number of flies unable to stand at 2-min intervals during exposure was counted. Time for 50% of each sample population of 25–30 flies to become sedated (ST50) was determined (panel A) and tolerance was quantified as the difference in ST50 between flies pre-exposed to ethanol and those pre-exposed to humidified air (panel B). There was a highly significant effect of genotype on tolerance, as indicated (***, p<0.001), and also of pre-exposure duration, as well as a significant interaction between genotype and pre-exposure duration (two-way ANOVA; n = 4 (30 min and 50 min pre-exposure) or 8 (40 min pre-exposure).
(TIF)

Figure S3 **The** ***intol*** **mutation is recessive.** Female flies (the *intol* mutation is X-linked) which were either *intol^+^/intol^+^* (Ctl), homozygous mutant (*intol/intol*) or heterozygous (*intol/+*) were pre-exposed to ethanol vapor (30 min at 60/40 relative flow rate; darker bars, left panel) or humidified air (lighter bars, left panel), allowed to recover for 3.5 hr, and assayed in the inebriometer. Tolerance was quantified as the difference in MET between flies pre-exposed to ethanol vapor vs. humidified air (right panel). A tolerance defect was seen for *intol/intol* homozygous flies, while *intol/+* flies were indistinguishable from Ctl (***, p<0.001; n = 4 (Ctl), 6 (*intol/intol*) or 8 (*intol/+*)). No significant effect of genotype on initial sensitivity (MET of air pre-exposed flies) was detected in this experiment (p>0.3).
(TIF)

Figure S4 **Mutations in** ***dlg1*** **cause reduced chronic tolerance.** The *intol* mutant, and 2 other independently identified *dlg1* mutants, exhibit reduced chronic tolerance development compared to the genetic background strain, 2202U (“Ctl”) (*, p<0.05; ***, p<0.001; one-way ANOVA with post hoc Holm-Sidak test; n = 7 (Ctl), 6 (*NP0768*, *NP1102*, *EY05003*), 11 (*intol*) or 4 (*NP4134*)). Chronic tolerance was measured in flies which were pre-exposed overnight to a low, non-sedating concentration of ethanol vapor (10∶80 relative units ethanol vapor: humidified air) or to humidified air alone, essentially as previously described [6].
(TIF)

Figure S5 **Dlg1 mutant larvae show loss of DlgS97 while DlgA expression is intact.** (A) Protein expression detected by immunofluorescence at wild-type and mutant 3^rd^ instar larval NMJ. Muscle 4 at abdominal segment A3 of control and *dlg* mutant larvae was triple-labeled with anti-dSAP97 (DlgS97N), anti-pan-Dlg antibody (DlgPDZ) and Cy3-HRP (HRP). Each image represents a stack of 15 optical sections taken at 0.5 µm steps. (B) Western blot analysis of body wall muscles from the wild type control and the *dlg1* mutants *15779*, *intol*, *NP0768*, *NP1102*, and *NP4314*. Immunoblot analysis of protein from 3^rd^ instar larvae was performed using anti-pan-Dlg antibody (DlgPDZ) and DlgS97-specific antibody (DlgS97N).
(TIF)

Figure S6 **Hypomorphic mutants of** ***Nmdar1*** **exhibit reduced chronic ethanol tolerance.** Ethanol sensitivity (A) and chronic tolerance (B) were quantified using the inebriometer for 2 different homozygous insertion mutants in *Nmdar1*, *EP331* and *EP3511*, compared to isogenic background control flies. The *EP3511* mutant showed a highly significant reduction in tolerance, and also exhibited increased sensitivity (p<0.001, one-way ANOVA with post hoc Holm-Sidak; n = 9 or 10). A second mutant in *Nmdar1*, *EP331*, also showed significantly reduced tolerance (p<0.001), but no alteration in sensitivity (n = 9). In panel A, dark colored bars represent inebriometer METs of flies pre-exposed to ethanol vapor, and light bars represent METs of parallel samples for each genotype pre-exposed to humidified air.
(TIF)

Methods S1 (DOC)
