# Supplementary figures and images for: DlgS97/SAP97, a Neuronal Isoform of Discs Large, Regulates Ethanol Tolerance
Source: PLoS One. 2012 Nov 7;7(11):e48967. doi: 10.1371/journal.pone.0048967 (PMC3492131; doi:10.1371/journal.pone.0048967)

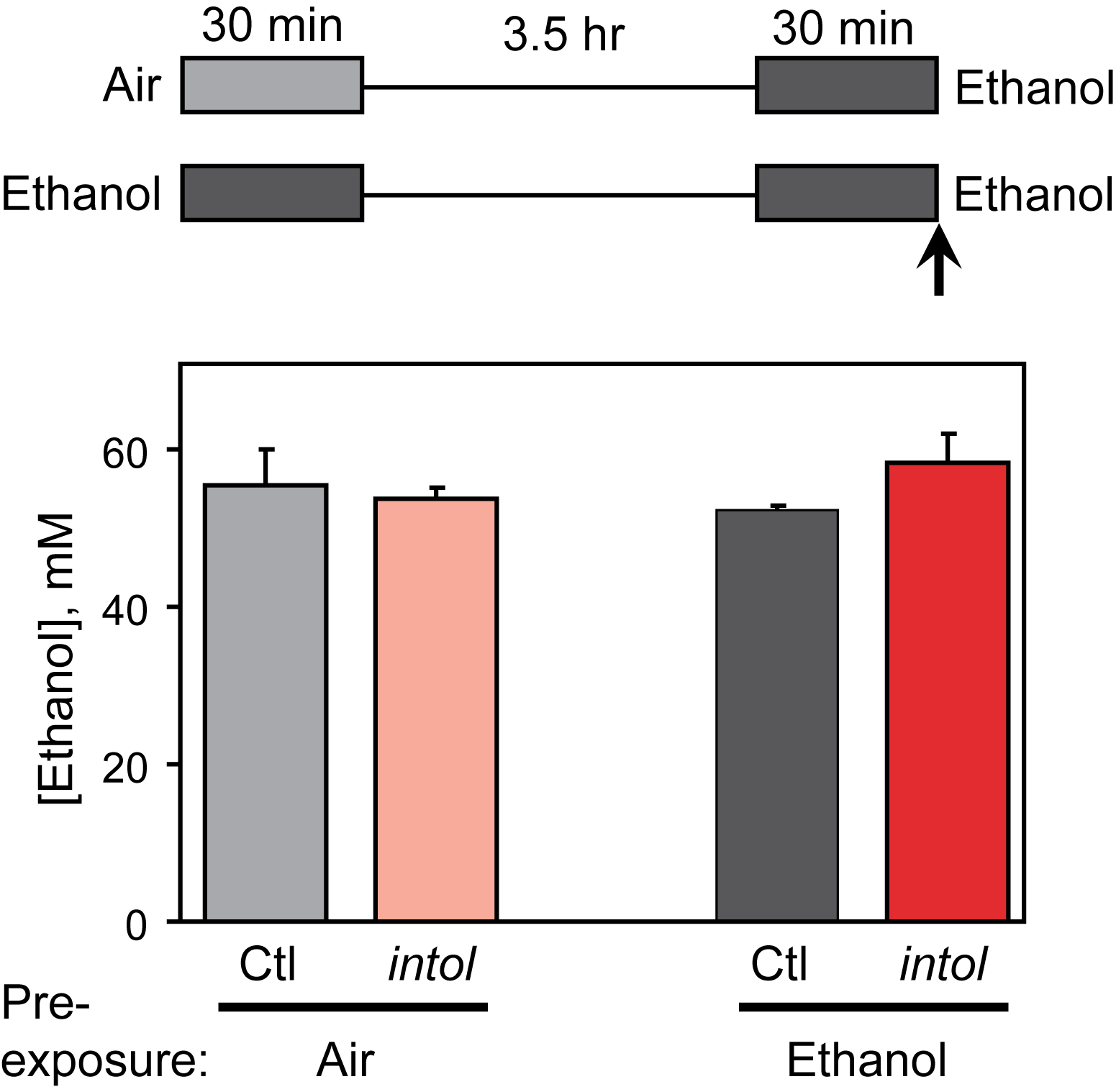

Supplement: Figure S1 — The intolerant mutant flies exhibit normal ethanol absorption and metabolism. Control (2202U) and intol mutant flies were grown and collected as for behavioral experiments, pre-exposed to ethanol vapor or humidified air, allowed to recover and re-exposed all to ethanol vapor, and snap-frozen at time indicated by arrow in schematic. Frozen flies were processed and ethanol content quantified. No significant effect of either genotype or pre-exposure condition (air vs. ethanol vapor) was seen on ethanol content of flies, nor was there a significant interaction between genotype and pre-exposure condition (two-way ANOVA; n = 4). (TIF) [file pone.0048967.s001.tif]

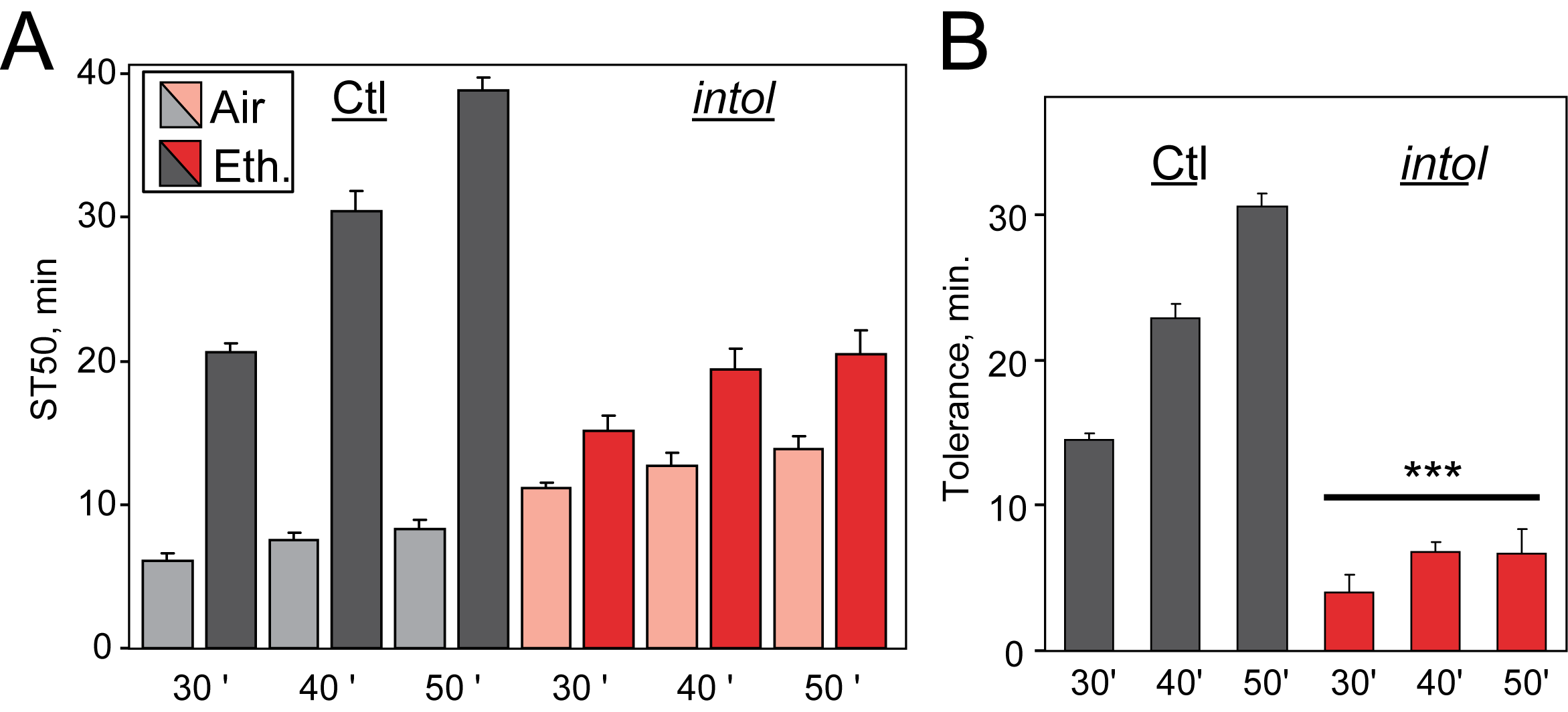

Supplement: Figure S2 — A longer ethanol pre-exposure does not correct the intol mutant tolerance deficit. Parental (2202U) and intol mutant flies were pre-exposed to ethanol vapor or to humidified air (control) for 30, 40 or 50 min as indicated and allowed to recover for ∼3.5 hr. All samples were then exposed to ethanol vapor (110∶40 relative flow ethanol vapor:humidified air), and the number of flies unable to stand at 2-min intervals during exposure was counted. Time for 50% of each sample population of 25–30 flies to become sedated (ST50) was determined (panel A) and tolerance was quantified as the difference in ST50 between flies pre-exposed to ethanol and those pre-exposed to humidified air (panel B). There was a highly significant effect of genotype on tolerance, as indicated (***, p<0.001), and also of pre-exposure duration, as well as a significant interaction between genotype and pre-exposure duration (two-way ANOVA; n = 4 (30 min and 50 min pre-exposure) or 8 (40 min pre-exposure). (TIF) [file pone.0048967.s002.tif]

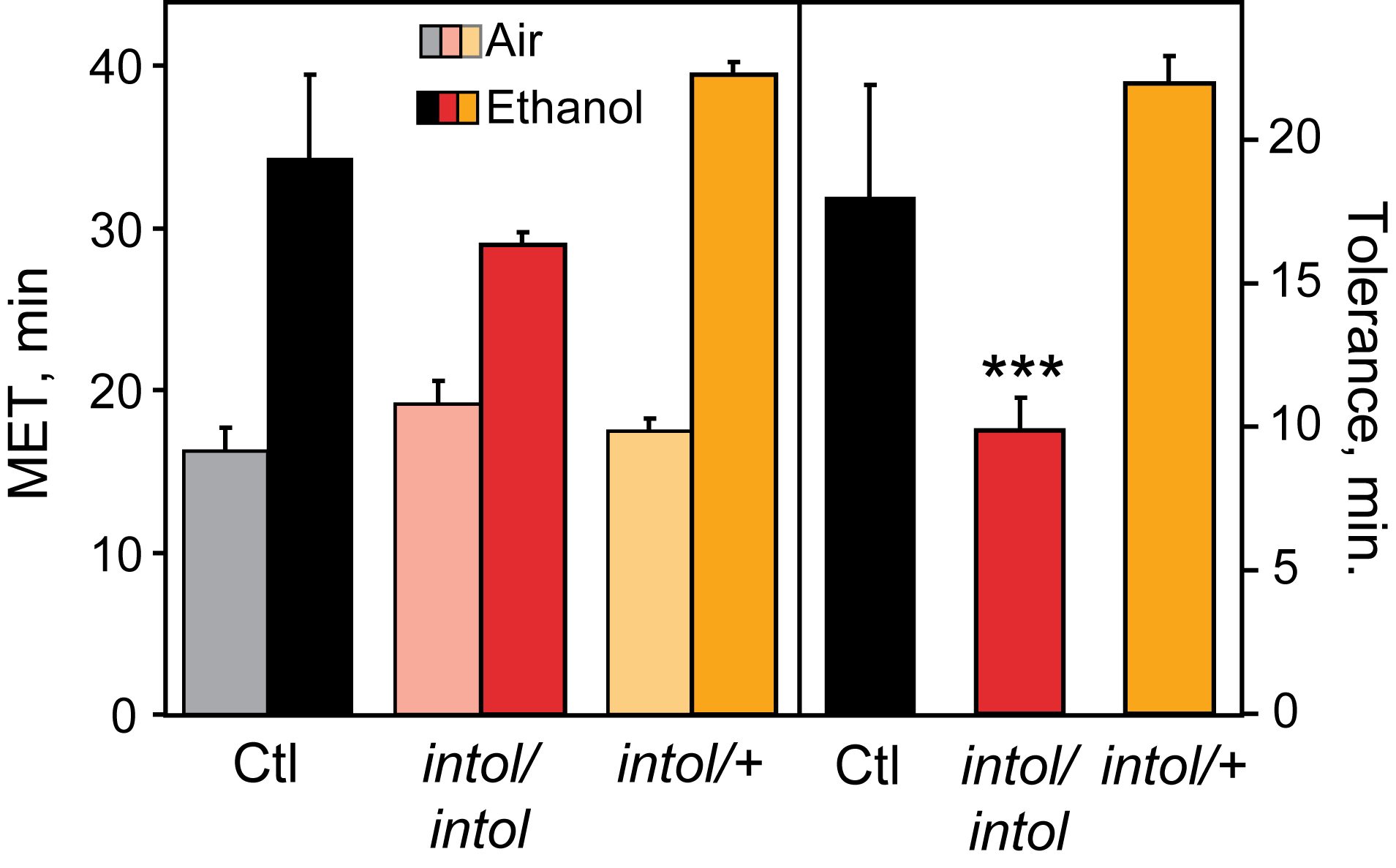

Supplement: Figure S3 — The intol mutation is recessive. Female flies (the intol mutation is X-linked) which were either intol+/intol+ (Ctl), homozygous mutant (intol/intol) or heterozygous (intol/+) were pre-exposed to ethanol vapor (30 min at 60/40 relative flow rate; darker bars, left panel) or humidified air (lighter bars, left panel), allowed to recover for 3.5 hr, and assayed in the inebriometer. Tolerance was quantified as the difference in MET between flies pre-exposed to ethanol vapor vs. humidified air (right panel). A tolerance defect was seen for intol/intol homozygous flies, while intol/+ flies were indistinguishable from Ctl (***, p<0.001; n = 4 (Ctl), 6 (intol/intol) or 8 (intol/+)). No significant effect of genotype on initial sensitivity (MET of air pre-exposed flies) was detected in this experiment (p>0.3). (TIF) [file pone.0048967.s003.tif]

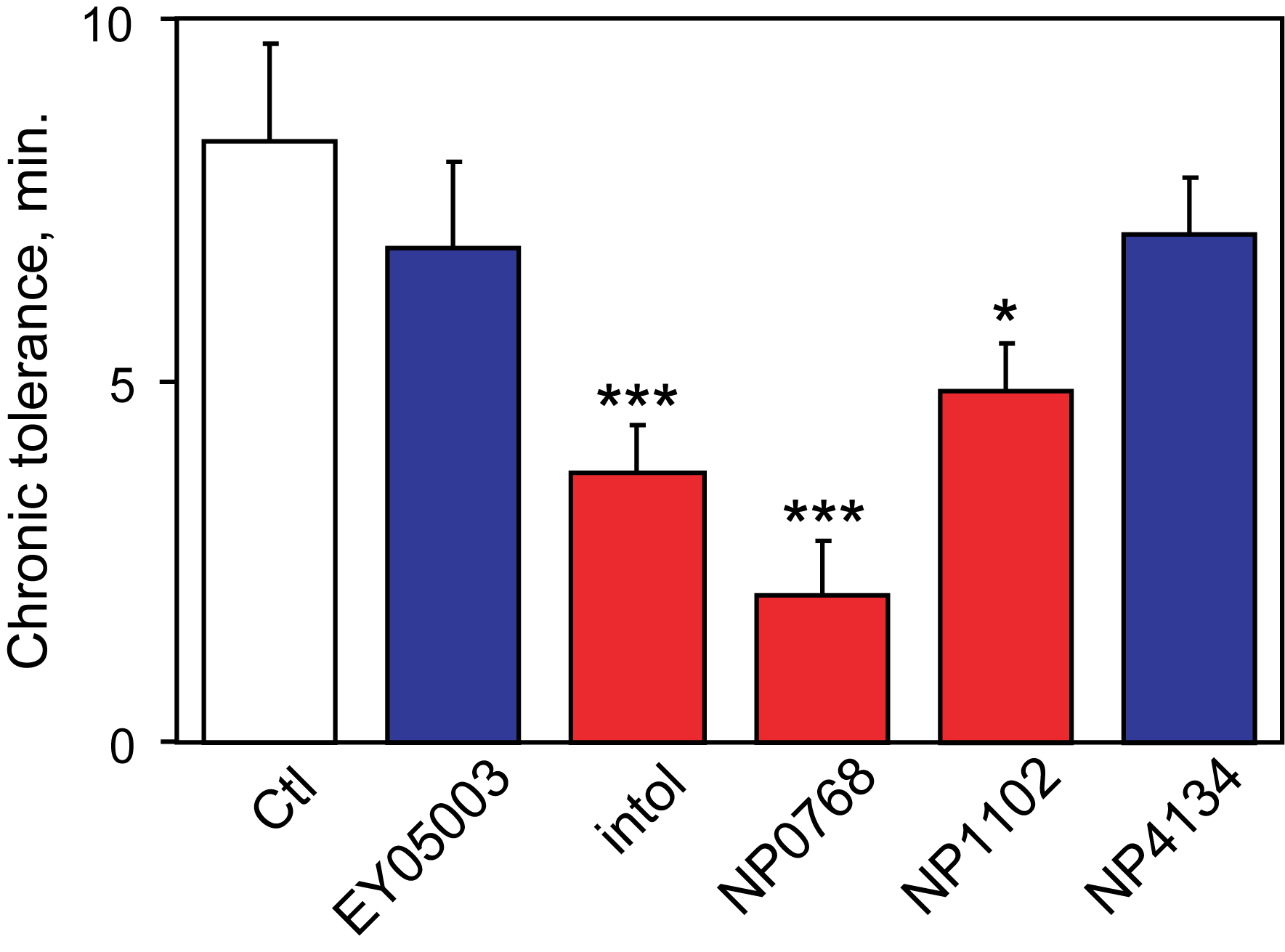

Supplement: Figure S4 — Mutations in dlg1 cause reduced chronic tolerance. The intol mutant, and 2 other independently identified dlg1 mutants, exhibit reduced chronic tolerance development compared to the genetic background strain, 2202U (“Ctl”) (*, p<0.05; ***, p<0.001; one-way ANOVA with post hoc Holm-Sidak test; n = 7 (Ctl), 6 (NP0768, NP1102, EY05003), 11 (intol) or 4 (NP4134)). Chronic tolerance was measured in flies which were pre-exposed overnight to a low, non-sedating concentration of ethanol vapor (10∶80 relative units ethanol vapor: humidified air) or to humidified air alone, essentially as previously described [6]. (TIF) [file pone.0048967.s004.tif]

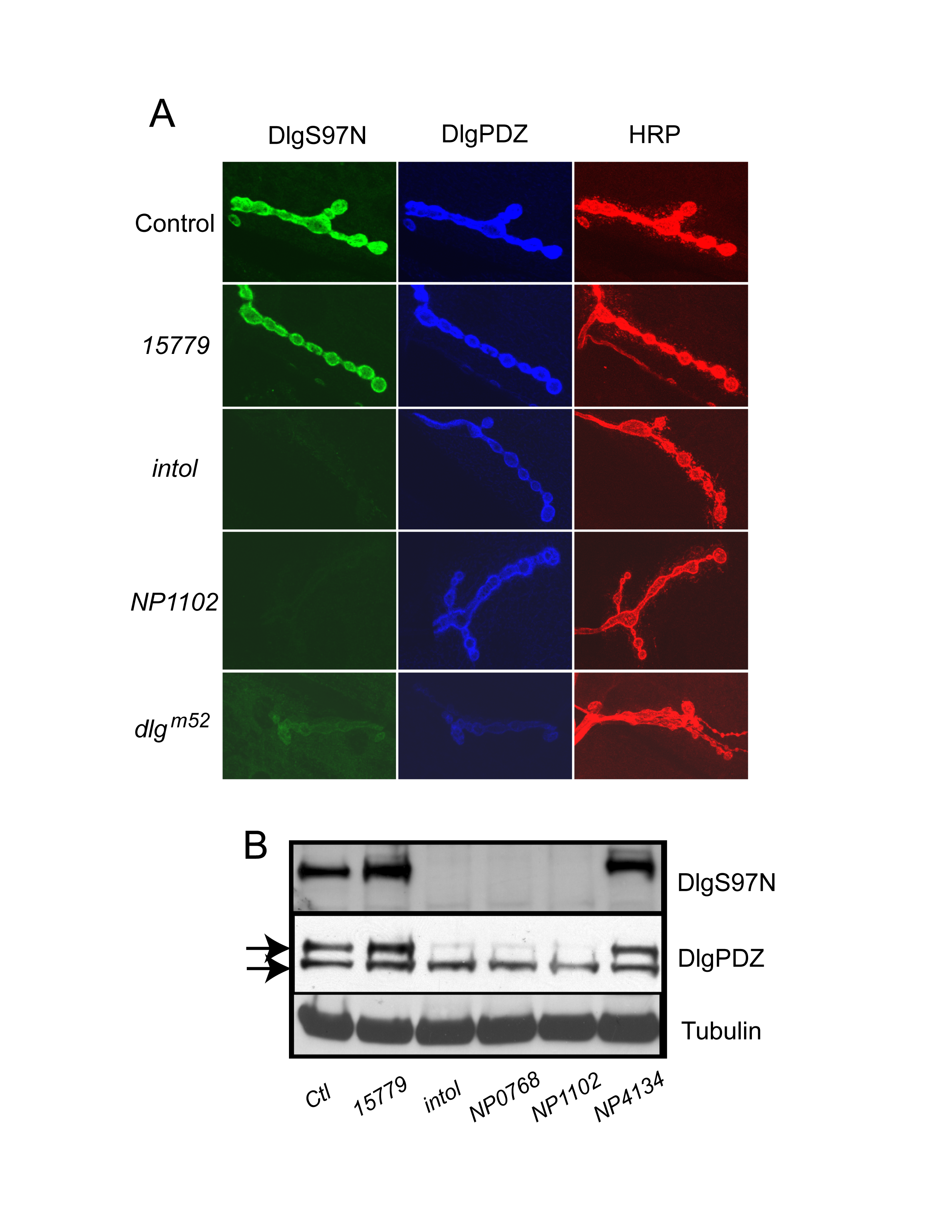

Supplement: Figure S5 — Dlg1 mutant larvae show loss of DlgS97 while DlgA expression is intact. (A) Protein expression detected by immunofluorescence at wild-type and mutant 3rd instar larval NMJ. Muscle 4 at abdominal segment A3 of control and dlg mutant larvae was triple-labeled with anti-dSAP97 (DlgS97N), anti-pan-Dlg antibody (DlgPDZ) and Cy3-HRP (HRP). Each image represents a stack of 15 optical sections taken at 0.5 µm steps. (B) Western blot analysis of body wall muscles from the wild type control and the dlg1 mutants 15779, intol, NP0768, NP1102, and NP4314. Immunoblot analysis of protein from 3rd instar larvae was performed using anti-pan-Dlg antibody (DlgPDZ) and DlgS97-specific antibody (DlgS97N). (TIF) [file pone.0048967.s005.tif]

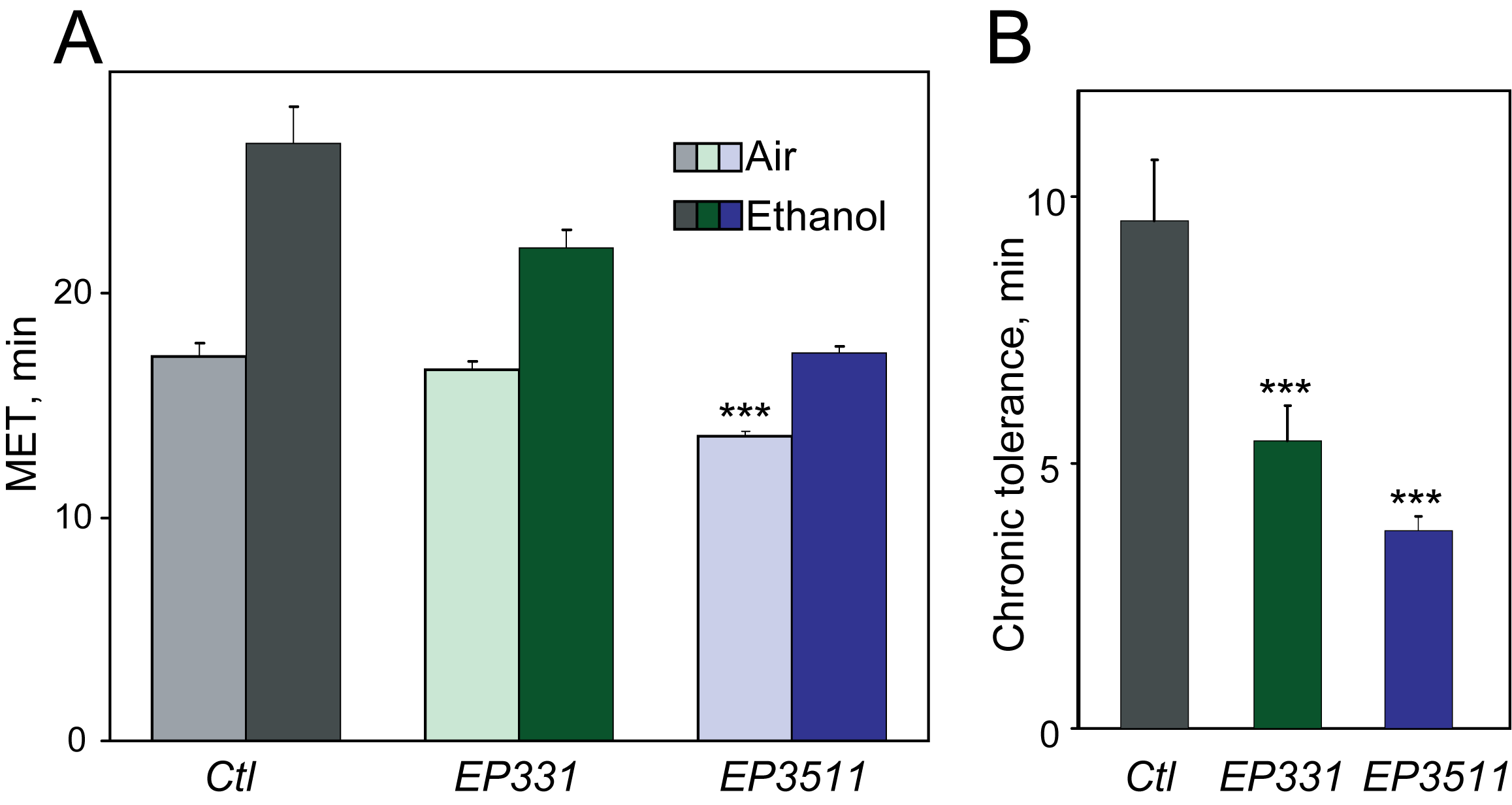

Supplement: Figure S6 — Hypomorphic mutants of Nmdar1 exhibit reduced chronic ethanol tolerance. Ethanol sensitivity (A) and chronic tolerance (B) were quantified using the inebriometer for 2 different homozygous insertion mutants in Nmdar1, EP331 and EP3511, compared to isogenic background control flies. The EP3511 mutant showed a highly significant reduction in tolerance, and also exhibited increased sensitivity (p<0.001, one-way ANOVA with post hoc Holm-Sidak; n = 9 or 10). A second mutant in Nmdar1, EP331, also showed significantly reduced tolerance (p<0.001), but no alteration in sensitivity (n = 9). In panel A, dark colored bars represent inebriometer METs of flies pre-exposed to ethanol vapor, and light bars represent METs of parallel samples for each genotype pre-exposed to humidified air. (TIF) [file pone.0048967.s006.tif]
