# Supplementary material for: DlgS97/SAP97, a Neuronal Isoform of Discs Large, Regulates Ethanol Tolerance
Source: PLoS One. 2012 Nov 7;7(11):e48967. doi: 10.1371/journal.pone.0048967 (PMC3492131; doi:10.1371/journal.pone.0048967)
Supplement: Methods S1 — (DOC) [file pone.0048967.s007.doc]

**Methods S1**

**Ethanol Absorption and Metabolism:** Fly internal ethanol concentrations were determined from whole fly homogenates of 25 flies per sample. Flies were exposed to ethanol vapor in perforated 50-mL Falcon tubes and snap-frozen on dry ice immediately following the final ethanol exposure. Samples were either stored at 80^o^C or homogenized immediately at 4^o^C in 250 μL of 50mM Tris–HCl, pH 7.5. Homogenates were centrifuged at 15,000x g for 20 minutes at 4^o^C and ethanol concentrations in supernatants were measured using an alcohol dehydrogenase-based spectrophotometric assay (Ethanol Assay Kit #229-29; Diagnostics Chemicals Limited, Oxford, CT). To calculate fly internal ethanol concentration, the volume of 1 fly was estimated to be ~2 μL; there was no obvious strain-based difference in fly volumes (sizes).

**Ethanol Sedation Assay:** Assays to measure sedation of flies by ethanol were performed in the Booz-o-mat essentially as described [37]. For each assay, up to 8 samples of 25-30 flies were scored at 2-min intervals of exposure to ethanol vapor, by twirling the perforated sample tubes containing the flies, then counting and scoring the flies unable to right themselves as sedated. The time at which 50% of the flies in a sample population were scored as sedated (ST50) was calculated using SigmaPlot (Systat Software, Inc.). Tolerance was quantified as the difference in ST50 between flies previously exposed to ethanol and control flies previously exposed to humidified air.

**Chronic tolerance:** Chronic tolerance was measured in the inebriometer following overnight (~16-20 hr) exposure of flies to either a low concentration of ethanol vapor (10/80 ethanol/air relative units) or to humidified air alone at an equivalent total airflow, essentially as previously described [6].
